# Supplementary material for: Investigation of Physicochemical Properties of the Structurally Modified Nanosized Silicate-Substituted Hydroxyapatite Co-Doped with Eu3+ and Sr2+ Ions
Source: Nanomaterials (Basel). 2020 Dec 24;11(1):27. doi: 10.3390/nano11010027 (PMC7823659; doi:10.3390/nano11010027)
Supplement: Supplementary file 1 [file nanomaterials-11-00027-s001.pdf]

nanomaterials

Supplementary Materials

Investigation of Physicochemical Properties of the Structurally Modified Nanosized Silicate-Substituted Hydroxyapatite Co-Doped with Eu^{3+} and Sr^{2+} ions

Sara Targonska * and Rafal J. Wiglusz *

Institute of Low Temperature and Structure Research, Polish Academy of Sciences, Okolna 2, 50-422 Wroclaw, Poland

* Correspondence: s.targonska@intibs.pl (S.T.); r.wiglusz@intibs.pl (R.J.W.); Tel.: +48-071-3954-159-274 (R.J.W.)

Table S1. The number of substrates used for synthesis of silicate-substituted hydroxyapatite co-doped with Eu^{3+} and Sr^{2+}.

Sample / Substrate	$Ca_{9.8-x}Sr_{0.2}Eu_x(PO_4)_2(SiO_4)_4(OH)_2$		
	**0.5 mol%**	**1.0 mol%**	**2.0 mol%**
**$Ca(NO_3)_2 \cdot 4H_2O$ [g]**	4.568	4.519	4.424
**$(NH_4)_2HPO_4$ [g]**	0.524	0.521	0.515
**tetraethyl orthosilicate TEOS [mL]**	1.76	1.75	1.73
**Eu_2O_3 [g]**	0.017	0.035	0.069
**$Sr(NO_3)_2$ [g]**	0.084	0.083	0.083

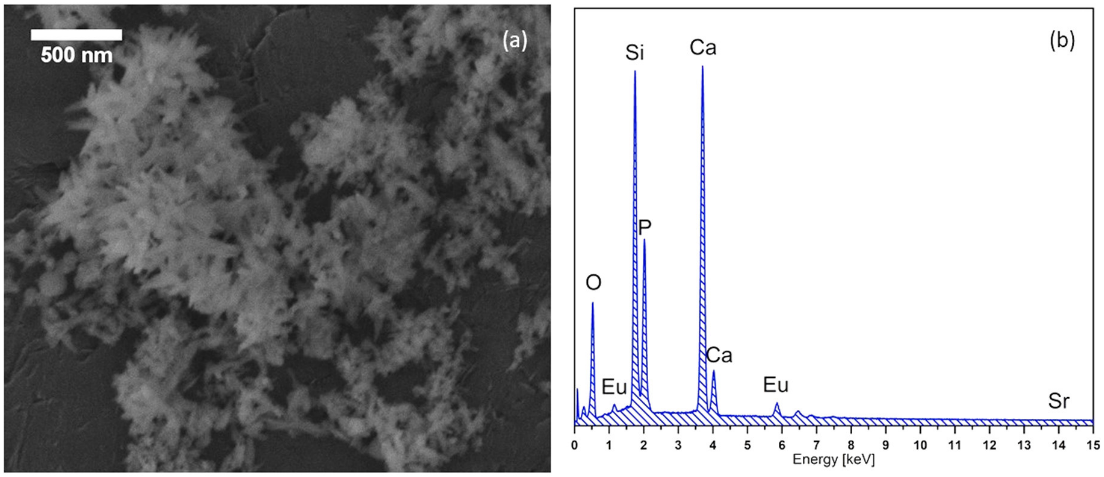

Figure S1. The representative SEM image (**a**) and EDS spectra (**b**) of the $Ca_{9.6}Sr_{0.2}Eu_{0.2}(PO_4)_2(SiO_4)_4(OH)_2$ nanopawders.

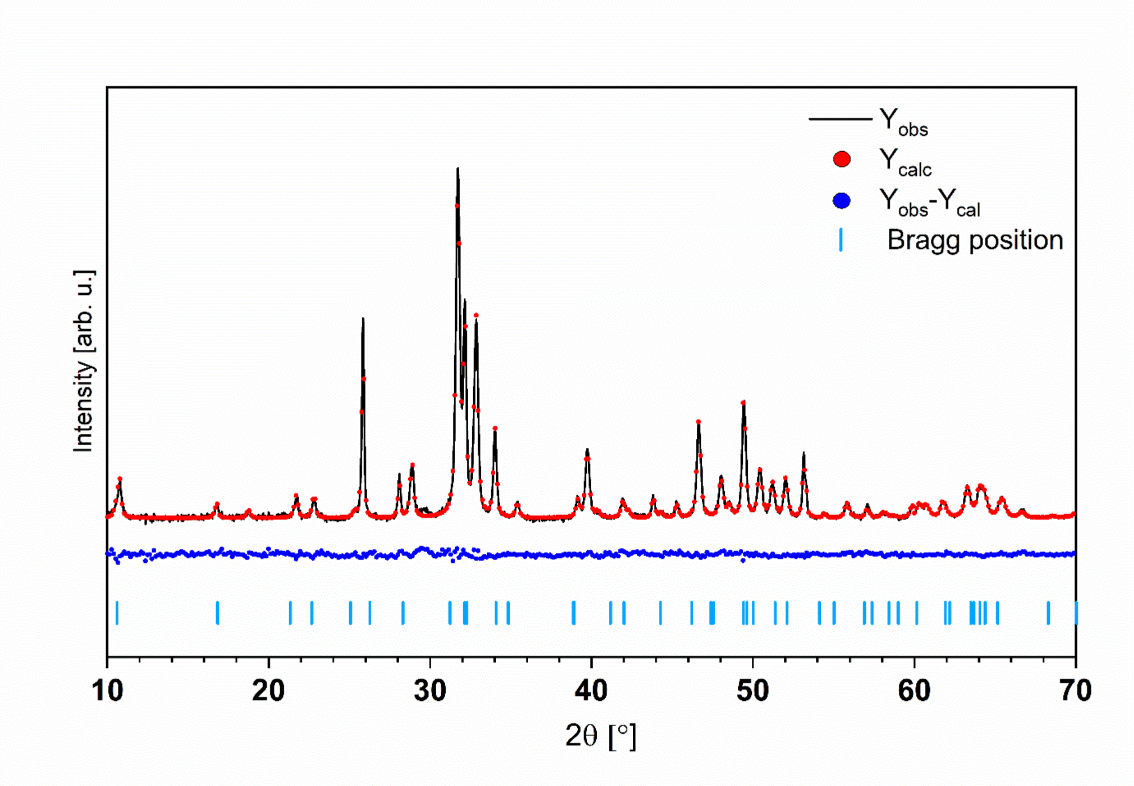

Figure S2. Representative results of the of the $Sr_{0.2}Eu_{0.2}Ca_{9.6}(PO_4)_2(SiO_4)_4(OH)_2$, obtained at 600°C, Rietveld analysis (red – fitted diffraction; blue – differential pattern, column – reference phase peak position).

Table S2. Unit cell parameters (a,c), cell volume (V), grain size as well as refine factor (R_w) for the $Ca_{10}(PO_4)_2(SiO_4)_4(OH)_2$ co-doped with 2 mol% Sr^{2+} and x mol/% Eu^{3+} ions (where x = 0.5 – 2).

Sample	a (Å)	c (Å)	V (Å^3)	size (nm)	R_w (%)
**single crystal** [1]	9.4106(2)	6.9166(2)	530.47(2)	–	–
			**600°C**		
**x = 0.5**	9.4343(2)	6.8860(3)	530.78(8)	54.66	2.0
**x = 1**	9.4288(0)	6.8900(1)	530.40(4)	56.00	2.5
**x = 2**	9.4231(3)	6.8858(6)	529.51(6)	45.37	2.1
			**$Sr_{0.2}Eu_{0.2}Ca_{9.6}(PO_4)_4(SiO_4)_2(OH)_2$**		
**as prepared**	9.4387(8)	6.8897(1)	531.57(3)	16.12	1.9
**400°C**	9.4291(6)	6.8902(7)	530.53(4)	16.61	1.7
**500°C**	9.4307(1)	6.8871(0)	530.46(4)	35.77	2.4
**600°C**	9.4231(3)	6.8858(6)	529.51(6)	45.37	2.1

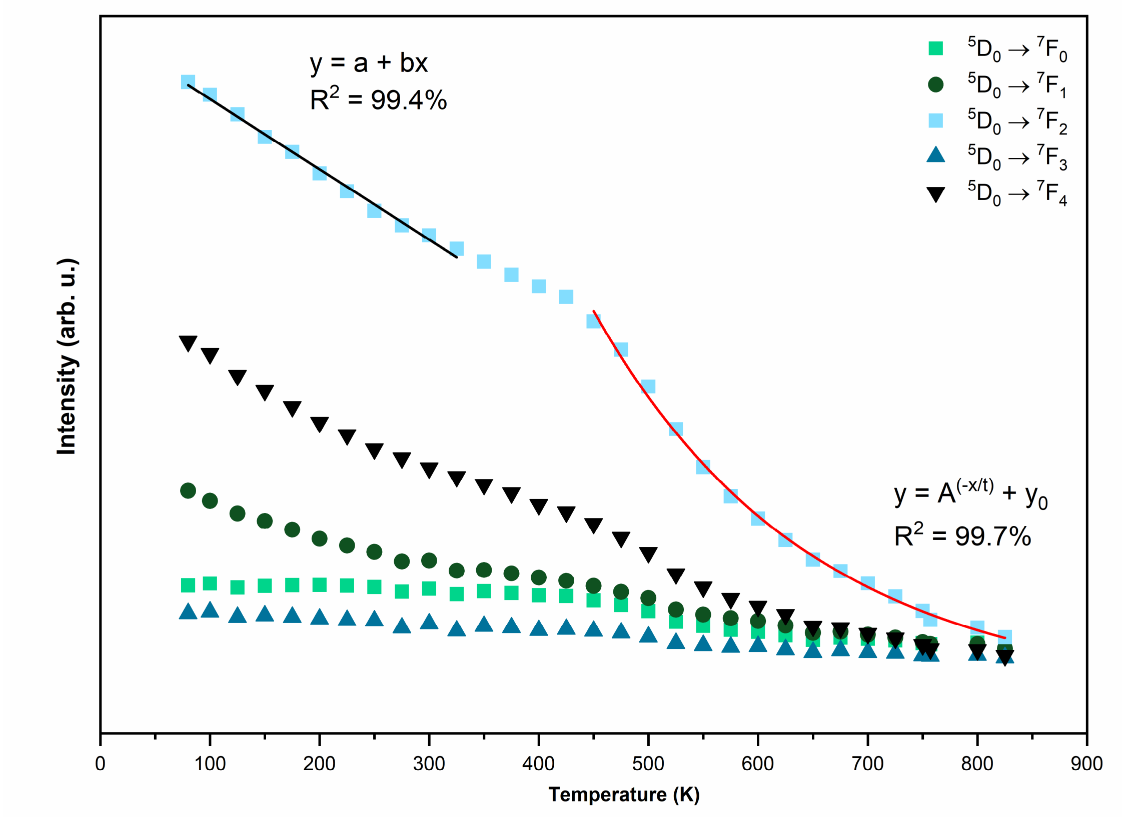

Figure S3. Temperature-dependent emission intensity of the lines correspond to the listed transitions.

Table S3. The comparison of the CIE color coordinates (x,y) of $Ca_{9.6}Sr_{0.2}Eu_{0.2}(PO_4)_2(SiO_4)_4(OH)_2$ as a function of ambient temperature.

Temperature (K)	CIE (x)	CIE (y)	Colour
80	0.643	0.356	Reddish orange
100	0.644	0.356	
125	0.645	0.354	
150	0.645	0.355	
175	0.645	0.354	
200	0.645	0.354	
225	0.645	0.355	
250	0.645	0.355	
275	0.646	0.353	
300	0.644	0.355	
325	0.647	0.353	
350	0.645	0.355	
375	0.645	0.355	
400	0.645	0.354	
425	0.644	0.355	
450	0.644	0.356	
475	0.643	0.357	
500	0.641	0.359	
525	0.641	0.359	
550	0.638	0.362	
575	0.634	0.366	
600	0.630	0.370	
625	0.627	0.372	
650	0.626	0.373	
675	0.621	0.378	
700	0.618	0.382	
725	0.613	0.386	
750	0.609	0.391	Orange
757	0.603	0.397	
800	0.597	0.402	
825	0.590	0.409	
